# Distribution characteristics of selenium, cadmium and arsenic in rice grains and their genetic dissection by genome-wide association study

**DOI:** 10.3389/fgene.2022.1007896

**Published:** 2022-10-13

**Authors:** Wenxi Wang, Fan Zhang, Dapu Liu, Kai Chen, Bin Du, Xianjin Qiu, Jianlong Xu, Danying Xing

**Affiliations:** ^1^ College of Economy and Management, Hubei University of Technology, Wuhan, China; ^2^ MARA Key Laboratory of Sustainable Crop Production in the Middle Reaches of the Yangtze River (Co-construction by Ministry and Province), College of Agriculture, Yangtze University, Jingzhou, China; ^3^ Institute of Crop Science, Chinese Academy of Agricultural Sciences, Beijing, China; ^4^ Shenzhen Branch, Guangdong Laboratory for Lingnan Modern Agriculture, Agricultural Genomics Institute at Shenzhen, Chinese Academy of Agricultural Sciences, Shenzhen, China; ^5^ National Nanfan Research Institute (Sanya), Chinese Academy of Agricultural Sciences, Sanya, China

**Keywords:** rice, selenium, cadmium, arsenic, genome-wide association study, quantitative trait locus/loci (QTL), candidate gene

## Abstract

High selenium (Se) and low cadmium (Cd) and arsenic (As) contents in rice grains were good for human health. The genetic basis and relationship of Se, Cd and As concentrations in rice grains are still largely unknown. In the present study, large variations were observed in Se, Cd and As concentrations in brown and milled rice in normal and Se treatment conditions in 307 rice accessions from 3K Rice Genomes Project. Se fertilizer treatment greatly increased Se concentrations but had no obvious changes in concentrations of Cd and As both in brown and milled rice. Total of 237 QTL were identified for Se, Cd and As concentrations in brown and milled rice in normal and Se treatment conditions as well as ratio of concentrations under Se treatment to normal conditions. Only 19 QTL (13.4%) were mapped for concentrations of Se and Cd, Se and As, and Se, Cd and As in the same or adjacent regions, indicating that most Se concentration QTL are independent of Cd and As concentration QTL. Forty-three favorable alleles were identified for 40 candidate genes by gene-based association study and haplotype analysis in 14 important QTL regions. Se-enriched rice variety will be developed by pyramiding favorable alleles at different Se QTL and excluding undesirable alleles at Cd and As QTL, or combining favorable alleles at Se QTL with the alleles at Se-sensitive QTL by marker-assisted selection.

## Introduction

Selenium (Se) is an essential element for humans, which is important component for many enzymes in our body, and has immune function (Meplan and Hughes, 2020). Se deficiency would damage our health, such as Keshan disease, Kashin-Beck disease and cancer (Tapiero et al., 2003; Cox and Bastiaans, 2007). It is mainly taken up by food, and the recommended daily intake of Se is 50–200 μg day^−1^ (Gailer, 2009). In some area of China, Se concentration in soil is very low, resulting in the Se deficiency in human diet (Tapiero et al., 2003), and the average daily intake of Se in this area is 26–32 μg (Chen et al., 2002). On the other hand, with the intensive anthropogenic activities during last century such as metal mining, smelting, industrial activities and use of excess fertilizers (Bolan et al., 2013), two heavy metals, cadmium (Cd) and arsenic (As), have been large problems and become a growing concern (Feng et al., 2013). They are mainly taken up by food and water, and exceeded intake of them would cause diseases such as renal dysfunction, osteoporosis and cancer (Rikans and Yamano, 2000; Liu et al., 2007; Akesson et al., 2014). Because of soil and water contamination, the concentrations of these two elements exceeded the Chinese maximum permissible concentrations of them in rice grains in some areas in southern China (Shang et al., 2012; Du et al., 2013; Zhu et al., 2016; Wang et al., 2019).

Rice is one of the most important staple crops, and provides food for two-thirds of world’s population, especially for Chinese people (Zhang J. et al., 2018), thus it is also one of the main resources of above three elements. Biofortification is an effective way to improve Se concentration in rice grains. However, many variables are involved in Se biofortification strategies, such as the Se administration mode (soil fertilization, foliar spray, or hydroponics), Se dose, species and fertilizer form, crop species, and variety and growth stage. Foliar application is a valid alternative for Se enrichment of agricultural products. Besides, it was reported that Se can inhibit the uptake of heavy metals in plant including Cd and As (Feng et al., 2013) and reduced harmful effect of Cd on plants (Pedrero et al., 2008). Compared to Se fertilization to the soil, foliar application by-passes any interference due to soil chemistry and microbiology issues, ensuring a higher efficacy even with low volumes of Se solution (D'Amato et al., 2020). Eventually, it is important and fundamental to solve the problem by developing new variety with rich Se concentration but minimum Cd and As accumulations in rice grains, both in brown rice and milled rice.

The concentrations of Se, Cd and As in brown rice range from 0.025 to 7.500 mg kg^−1^, 0.002–0.475 mg kg^−1^, and 0.010–0.750 mg kg^−1^ in rice germplasms, respectively (Zhang et al., 2010; Wang et al., 2012; Kuramata et al., 2013; Zhang G. M. et al., 2018; Liu et al., 2019; Norton et al., 2019). All of them varied among different rice varieties (Zou et al., 2018), and belong to quantitative traits controlled by multiple genes (Zhang G. M. et al., 2018). Using molecular marker technology, some QTL controlling concentrations of Se, Cd and As in brown rice have been identified. For Se concentration, two QTL were identified on chromosome 5 using an F_2_ population derived from Xinfenghongmi and Minghui 100 (Zhang et al., 2010). For Cd, more than 35 QTL were detected on all 12 chromosomes using different populations (Li et al., 2020; Pan et al., 2021). For As concentration, three QTL were identified on chromosomes 6 and 8 using an F_2_ population for methylated arsenic (Kuramata et al., 2013), and two QTL for As accumulation were identified using an doubled haploid population (Zhang et al., 2008). Till now, only six genes including *OsHMA2*, *OsHMA3*, *OsLCT1*, *CAL1*, *OsCd1* and *OsNAMP5* have been cloned for Cd concentration in brown rice (Chen et al., 2019; Yan et al., 2019; Chang et al., 2020).

In recent years, a new approach named genome-wide association study (GWAS) was used for identifying QTL for rice complex traits. It is based on rice germplasm and linkage disequilibrium (LD). Using this strategy, some QTL for Se, Cd and As have been detected, and some candidate genes were identified. Liu *et al.* (2019) identified 17 and 22 QTL for Cd and As concentrations in rice grains, respectively in 276 accessions using 416K SNP genotypes. Norton et al. (2019) detected 74 QTL for As concentration in rice grains in 266 landraces using 2M SNP genotypes. Among them, six QTL were stably expressed across different environments. Zhang G. M. et al. (2018) identified 3 and 62 QTL for Se and Cd concentrations in rice grains, respectively, in 698 *xian* and *geng* germplasms using 27K SNP genotypes. Further, 198 candidate genes for Cd concentration were detected using 2.9M SNP genotypes. Yan et al. (2019) detected 12 QTL for Cd accumulation in rice grains using 3M SNP genotypes in 127 rice cultivars, and then successfully cloned *OsCd1* for Cd accumulation. Qiu T. C. et al. (2021) identified 9 QTL for Se concentration in milled rice in 207 accessions using 50K SNPs.

In the present study, Se, Cd and As concentrations in brown and milled rice were measured under normal and Se fertilizer treatment conditions, in a diverse panel of 307 accessions from 3K RGP (Wang et al., 2018). Based on analysis of distribution and relationship of the three elements in brown and milled rice under the two conditions, QTL for Se, Cd and As concentrations in brown and milled rice were identified by GWAS using high-quality SNPs from 3K GRP. Then, gene-based association studies were performed in some important QTL regions using all available SNPs from RFGB v2.0 database (Wang et al., 2020). Finally, haplotype analysis was used to identify the candidate genes and novel alleles. The aim in this study is to explore effects of Se fertilizer application on concentrations of Se, Cd and As and the relationships among them both in brown and milled rice at phenotypic level and genetic mechanisms underlying the concentrations of them. Our results will help us better understand the genetic basis of Se, Cd and As concentrations in brown and milled rice and provide candidate genes and favorable alleles for rice breeding of high Se, low Cd and As concentration by marker-assisted selection (MAS).

## Materials and methods

### Association mapping panel

In our previous study, a total of 1,016 rice accessions from 3K RAP (Wang et al., 2018) were identified to flower normally in Jinghzou of Hubei province, China (Qiu et al., 2020). To avoid the negative effects of lodging and large difference of heading date on the concentrations of Se, Cd and As in grains, 307 rice accessions with plant height below 140 cm and heading date within 10 days difference were selected as materials in the present study. They were from 33 countries worldwide (Supplementary Table S1).

### Field experiment and evaluation of Se, Cd and As concentrations in grains

All accessions were grown in the summer seasons of 2019 and 2020 at the Experimental Farm of College of Agriculture, Yangtze University, Jingzhou City (30.2°N, 112.7°E), Hubei province, China. The Se, Cd and As concentrations in the soil were averagely 0.33, 0.27 and 7.15 mg kg^−1^ in the 2 years. Field experiment was carried out using a randomized complete design. Each accession was planted in three rows and ten plants per row with the density of 17 cm between plants and 20 cm between rows for two replications in two conditions, i.e., normal condition where field management was followed as local farmers’ practices, and Se treatment condition where Se fertilizer in diluent with 3.30 g Na_2_SeO_3_ dissolved in 40 kg water per 666.7 m^2^ was sprayed to rice plants at booting stage and other field management was completely same as the normal condition. At mature stage, seeds were bulk-harvested from each plot, and air-dried and stored for more than 3 months at room temperature before assessing of concentrations of selenium (Se), cadmium (Cd) and arsenic (As) in brown and milled rice.

Seeds of each plot were de-hulled into brown rice, and then polished into milled rice according to the method described previously (Qiu et al., 2015). Brown and milled rice were ground into flour for assessing the three element concentrations by atomic fluorescence spectrometry (AFS-230E, Beijing Jinsuokun Technology Developing Co. Ltd, China) after microwave digestion (ECH-2, Shanghai Sineo Microwave Chemistry Technology Co. Ltd, China) following the methods described as Zhang G. M. et al. (2018) and Liu et al. (2019). The evaluated traits included Se concentration in brown rice and milled rice in normal condition (SBN, SMN), Cd concentration in brown rice and milled rice in normal condition (CBN, CMN), As concentration in brown rice and milled rice in normal condition (ABN, AMN), Se concentration in brown rice and milled rice in treatment condition (SBT, SMT), Cd concentration in brown rice and milled rice in treatment condition (CBT, CMT), and As concentration in brown rice and milled rice in treatment condition (ABT, AMT). To reflect effects of Se fertilizer addressing on concentrations of Se, Cd and As in rice grains, ratio of Se concentration in brown rice (RSB) and milled rice (RSM) under Se treatment to normal conditions, ratio of Cd concentration in brown rice (RCB) and milled rice (RCM) under Se treatment to normal conditions, and ratio of As concentration in brown rice (RAB) and milled rice (RAM) under Se treatment to normal conditions were calculated. All concentrations were independently tested three times per sample, and the mean value was used as the trait value for data analysis. Then, Statistica 5.5 was used for analysis of statistical parameters and correlations among different traits (Qiu et al., 2017).

### SNP data

The 4.8 M SNP dataset of 3K RGP was download from the Rice SNP-Seek Database (http://snp-seek.irri.org) (Alexandrov et al., 2015). For structure analysis, PLINK 1.9 was used to obtain 73,162 independent SNPs (Purcell et al., 2007). The parameter was set as MAF >0.05 and missing data ratio <0.10. ADMIXTURE program and GCTA software were used to identify genetic structure and principal component (Alexander et al., 2009). For GWAS, PLINK 1.9 was also used to identify high quality SNPs with similar parameter setting as MAF >0.05 and missing data ratio <0.20.

### QTL detection for concentrations of three elements by GWAS

GWAS was conducted to identify QTL for Se, Cd and As concentrations, and ratio of Se, Cd and As concentrations of treatment to normal conditions in brown and milled rice using 405,150 high quality SNPs and trait values of the 307 accessions. The SVS software package v8.4.0 was used for association analysis (Qiu X. et al., 2021). The EMMAX (Efficient Mix-Model Association eXpedited) implementation of the single-locus mixed linear model was fitted to the marker dataset (Vilhjálmsson and Nordborg, 2013). The threshold was set as *p* < 1.0E-05. Since the LD decay of 3K RGP was about 200 kb (Wang et al., 2018), QTL located in the region of 100 kb each side at the peak SNPs and containing more than ten SNP with *p* value above threshold were considered as one QTL.

### Gene-based association mapping and haplotypes analysis

Gene-based association mapping and haplotype analysis were conducted to detect candidate genes and favorable alleles in the important QTL regions. QTL regions identified for the same trait in both years and/or for more than two different traits were considered as important QTL. QTL with peak SNP having lowest *p* value in the regions were further analyzed. Firstly, all genes located within 100 kb up and down the peak SNP were retrieved from the Rice Genome Annotation Project (http://rice.plantbiology.msu.edu/). Secondly, all SNPs located on these genes were retrieved from 32 M SNPs data generated from 3K RGP in the RFGB v2.0 database (Wang et al., 2020), and they were used for gene-based association analysis. The threshold was also set as–log_10_(P) > 5.0 (*p* < 1.0E-05). Thirdly, for each QTL region, all significant SNPs located in promoter region, 5′ UTR region, non-synonymous SNPs in the exon regions, splice region in intron and 3’ UTR region of each candidate gene were used for identifying haplotypes. Candidate genes were assigned by testing significant difference for relevant traits among major haplotypes (samples more than 8) for each important QTL by ANOVA with *p* < 0.01. When QTL containing cloned genes for element-related traits, the cloned genes were considered as only candidate gene (s) of the target QTL.

## Results

### Phenotypic variations in the natural population

A wide range of concentrations of Se, Cd and As in both brown and milled rice under normal, treatment and ratio of treatment to normal conditions reflected the substantial genotypic variations associated with concentrations of three elements in the panel of accessions (Table 1). Phenotypic variations had a similar trend in 2 years. Take the data in 2019 as example, SMN, CMN and AMN amounted to 48.8%, 42.9% and 57.3% of SBN, CBN and ABN, respectively, indicating that concentrations of the three elements in milled rice (endosperm) accounted for approximately half of total concentrations in brown rice. Averagely, SBT, SMT, CBT, CMT, ABT and AMT were 3.9, 5.0, 0.9, 1.0, 0.9, and 1.0 times as much as SBN, SMN, CBN, CMN, ABN and AMN, respectively, indicating that Se fertilizer treatment substantially improved Se concentrations but no obvious changes in concentrations of Cd and As both in brown and milled rice. The ratios of treatment to normal conditions for the three element concentrations in milled rice were higher than those in brown rice, suggesting that Se-treatment accelerated accumulation of the three elements in endosperm. This was also reflected by the fact that SMT, CMT and AMT amounted to 62.1%, 50.0% and 64.7% of SBT, CBT and ABT, respectively, meaning that Se-treatment much improved the proportions of the three elements in milled rice as compared with those in normal condition.

**TABLE 1:** Concentrations of Se, Cd and As in brown and milled rice in normal and treatment conditions and their ratios of treatment to normal conditions in 307 accessions.

Trait^a^	2019			2020		
	Mean ± SD	Range	CV (%)	Mean ± SD	Range	CV (%)
SBN (mg kg^−1^)	0.041±0.045	0.002–0.452	109.87	0.091±0.232	0.013–1.994	254.54
SMN (mg kg^−1^)	0.020±0.021	0–0.209	107.24	0.054±0.196	0.006–1.775	360.53
CBN (mg kg^−1^)	0.007±0.031	0.001–0.430	417.94	0.012±0.091	0.004–0.707	74.59
CMN (mg kg^−1^)	0.003±0.002	0–0.025	76.74	0.009±0.003	0.003–0.018	35.10
ABN (mg kg^−1^)	0.419±0.125	0.024–1.037	29.83	0.173±0.042	0.082–0.335	24.43
AMN (mg kg^−1^)	0.240±0.087	0.106–0.680	36.11	0.125±0.030	0.059–0.199	24.14
SBT (mg kg^−1^)	0.161±0.150	0.029–1.014	92.90	0.265±0.191	0.038–1.113	72.08
SMT (mg kg^−1^)	0.100±0.982	0–0.769	99.11	0.180±0.138	0–0.533	76.55
CBT (mg kg^−1^)	0.006±0.007	0.001–0.069	122.66	0.010±0.004	0.003–0.019	35.90
CMT (mg kg^−1^)	0.003±0.002	0.001–0.009	50.68	0.008±0.003	0–0.016	39.39
ABT (mg kg^−1^)	0.374±0.252	0.141–3.392	57.31	0.179±0.039	0.076–0.300	21.63
AMT (mg kg^−1^)	0.242±0.084	0.022–0.512	34.90	0.133±0.032	0–0.239	24.12
RSB	5.587±4.920	0.304–31.57	88.08	7.878±7.792	0.222–32.298	98.91
RSM	6.991±6.901	0.034–43.016	98.71	9.866±11.947	0.879–59.369	121.09
RCB	1.541±2.395	0.209–19.222	155.38	1.185±0.750	0.206–3.374	63.27
RCM	1.557±1.425	0.310–10.468	91.51	1.026±0.556	0.404–3.147	54.22
RAB	0.961±0.718	0.247–8.438	74.71	1.114±0.393	0.353–3.085	35.31
RAM	1.153±0.519	0.170–3.411	45.01	1.163±0.453	0.330–3.202	38.94

^a^ SBN, se concentration in brown rice in normal condition; SMN, se concentration in milled rice in normal condition; CBN, cd concentration in brown rice in normal condition; CMN, cd concentration in milled rice in normal condition; ABN, as concentration in brown rice in normal condition; AMN, as concentration in milled rice in normal condition; SBT, se concentration in brown rice in treatment condition; SMT, se concentration in milled rice in treatment condition; CBT, cd concentration in brown rice in treatment condition; CMT, cd concentration in milled rice in treatment condition; ABT, as concentration in brown rice in treatment condition; AMT, as concentration in milled rice in treatment condition; RSB, ratio of Se concentration in brown rice; RSM, ratio of Se concentration in milled rice; RCB, ratio of Cd concentration in brown rice; RCM, ratio of Cd concentration in milled rice; RAB, ratio of As concentration in brown rice; RAM, ratio of As concentration in milled rice.

Among the 307 accessions, three accessions (IRIS_313–8208, IRIS_313–8856 and IRIS_313–11968) were consistently identified to have high Se concentration, and low Cd and As concentrations in milled rice in normal condition in 2 years (Supplementary Table S2). Specifically, IRIS_313–8208 averagely had 0.067 mg kg^−1^ SMN, 0.005 mg kg^−1^ CMN and 0.161 mg kg^−1^ AMN; IRIS_313–8856 averagely had 0.108 mg kg^−1^ SMN, 0.004 mg kg^−1^ CMN and 0.189 mg kg^−1^ AMN; and IRIS_313–11968 averagely had 0.044 mg kg^−1^ SMN, 0.004 mg kg^−1^ CMN and 0.130 mg kg^−1^ AMN. IRIS_313–10083 had high Se concentration, and low Cd and As concentrations in milled rice in Se treatment condition in 2 years, with average 0.333 mg kg^−1^ SMT, 0.003 mg kg^−1^ CMT and 0.139 mg kg^−1^ AMT. Another four accessions (CX115, IRIS_313–11039, IRIS_313–11197 and IRIS_313–11943) were consistently identified as high ratio of Se concentration and low ratios of Cd and As concentrations in milled rice (Supplementary Table S2).

### Correlation of concentrations in the panel of accessions

We analyzed correlations of three element concentrations in different parts under different treatments (Supplementary Table S3). For the same element between brown and milled rice under normal and treatment conditions, all three elements showed consistently highly significant positive correlations between brown and milled rice both in 2 years, suggesting accessions with high concentrations of three elements in brown rice would had high concentrations in milled rice. Se and Cd concentrations had no significant correlation both in brown and milled rice in the 2 years under normal and treatment conditions except for a highly positive correlation in milled rice in 2020 under treatment condition, suggesting that Se was almost independent of Cd in milled and brown rice under normal condition. Se and As had highly significant negative correlations in brown and milled rice only in 2019 under normal condition, and highly significant negative correlation in milled rice under treatment condition in 2 years, indicating that Se was antagonistic to As in milled rice. There were no significant correlations between Cd and As in brown and milled rice under normal and treatment conditions in 2 years except that in milled rice in 2020 under treatment, indicating that Cd and As are mostly independent. Correlations of the same elements Se, Cd and As in the same parts were all no significant between normal and treatment conditions in brown rice in 2 years. In milled rice, however, correlation of Se and As was significant positive between normal and treatment conditions in 2019, indicating that Se and As were had consistent responses to Se treatment in milled rice than brown rice among different accessions.

### Detection of QTL by GWAS

**FIGURE 1:**
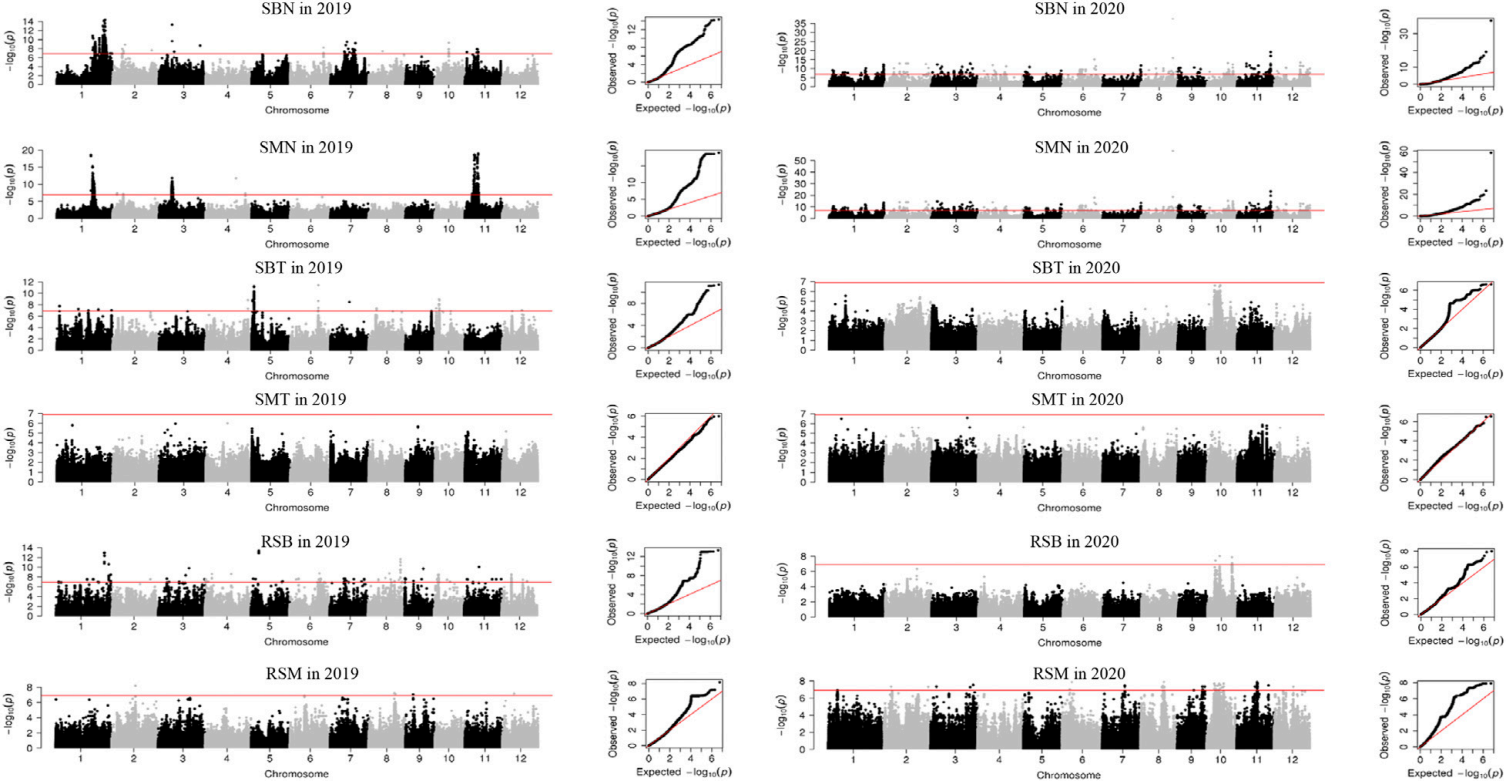
Manhattan plots and QQ plots of GWAS for Se concentration in 2019 and 2020 in the 307 rice accessions. SBN, Se concentration in brown rice in normal condition; SMN, Se concentration in milled rice in normal condition; SBT, Se concentration in brown rice in treatment condition; SMT, Se concentration in milled rice in treatment condition; RSB, ratio of the Se concentration in brown rice; RSM, ratio of the Se concentration in milled rice.

Three main subpopulations were identified by admixture analysis and principal component analysis (Supplementary Figure S1), including *xian* (*indica*, 138), *geng* (*japonica*, 132), *aus* (32). In addition, five accessions belong to *admix*. Using 405,150 high quality of 307 accessions, a total of 237 QTL were identified for 16 traits, ranging from four for AMT to 23 for RCB and RAB. Among them, three QTL were identified in both years (Supplementary Table S4; Figure 1; Supplementary Table S2). None QTL was detected for SMT and CMT.

In normal condition, 87 QTL were detected for six traits (Supplementary Table S4; Figure 1; Supplementary Table S2). For Se concentrations, 21 QTL for SBN and 18 for SMN were detected on all 12 chromosomes in 2 years, and explained phenotypic variations of 1.00%–18.69%. Among them, *qSMN11.1* was stably detected in both years. For Cd concentrations, 17 QTL for CBN and 13 QTL for CMN were detected on all 12 chromosomes with average phenotypic variation of 7.38% and a range from 1.01% to 41.35% in 2 years. Of them, two QTL (*qCBN5* and *qCBN9*) were stably detected in both years. For As concentration, nine QTL for each of ABN and AMN were identified on 12 chromosomes except chromosome 10 in 2019, accounting for phenotypic variation ranging from 1.01% to 23.84%.

A total of 55 QTL were identified for concentrations of three elements in treatment condition (Supplementary Table S4; Figure 1; Supplementary Table S2). Ten SBT QTL were detected on chromosomes 1, 2, 5, 6, 8–10, 12, with an average phenotypic variation of 11.12%. Nineteen CBT QTL were detected on all 12 chromosomes in 2019 with 1.45%–12.76% of phenotypic variations. For As concentration, 22 and 4 QTL for ABT and AMT were identified in 2019 and 2020, respectively. They explained phenotypic variation from 1.21% to 13.08%.

For ratio of concentrations of three elements under Se treatment to normal conditions, a total of 95 QTL were identified for RSB, RSM, RCB, RCM, RAB and RAM (Supplementary Table S4; Figure 1; Supplementary Table S2). Nineteen and 13 QTL for RSB and RSM were detected on all 12 chromosomes, respectively, explaining phenotypic variation of 1.79%–40.14%. Nineteen and 22 QTL were identified for RCB and RCM, respectively, with mean phenotypic variation of 4.14%, ranging from 1.21% to 12.76%. Twenty-three and five QTL were found for RAB and RAM on all chromosomes with phenotypic variation of 0.21%–18.37%.

### Genetic overlap among QTL underlying different elements in grains

Comparison of QTL located in the same or adjacent regions can reveal genetic relationships of the concentration between brown and milled rice and among three elements. Total of 46 regions harboring QTL for different traits were detected (Supplementary Table S5). Among them, 11 regions were identified for concentrations of the same element between brown rice and milled rice, including 10 harboring *qSBN2.2* and *qSMN2.1*, *qSBN2.3* and *qSMN2.2*, *qSBN3.1* and *qSMN3.1*, *qSBN4.2* and *qSMN4.2*, *qSBN5.2* and *qSMN5.2*, *qSBN6* and *qSMN6*, *qSBN7.2* and *qSMN7*, *qSBN8.2* and *qSMN8*, *qSBN10* and *qSMN10*, and *qSBN11.2* and *qSMN11.2* for Se concentration in brown and milled rice under normal conditions, and one harboring *qAMN2* and *qABN2* for As concentration in brown and milled rice under normal condition. It was indicated that pleiotropy is most probably responsible for association of Se and As concentrations between brown and milled rice. Seventeen regions were identified for concentrations of two or three elements, including three harboring *qSMN1.2* and *qCBN1.2*, *qSBN8.2*, *qSMN8* and *qCBN8.2*, and *qSBT12* and *qCBT12* affecting Se and Cd concentrations in normal or treatment conditions, 10 harboring *qCBN2*, *qABN2* and *qAMN2*, *qCBT2.1* and *qABT2.1*, *qCBN3*, *qCBT3.1* and *qABT3.2*, *qCBT3.2* and *qABT3.3*, *qCBT4.1*, *qABT4.1* and *qAMT4*, *qCBT7* and *qABT7*, *qCBT8.2* and *qABT8.3*, *qCBT9.1* and *qABT9.1*, *qCBT10* and *qABT10.1*, and *qCBN11* and *qAMN11* for Cd and As concentrations under normal or treatment condition, and four harboring *qSBT1.2*, *qCBT1.2* and *qABT1.2*, *qSBT5*, *qCBT5* and *qABT5*, *qSBT8*, *qCMN8* and *qABT8.1*, and *qSBT9*, *qCBT9.2* and *qABT9.2* affecting Se, Cd and As concentrations in brown rice under treatment or normal condition. Twenty regions were identified for QTL overlap between concentrations in normal or treatment conditions and concentration ratio of the three elements. It was indicated that partial genetic overlaps exist due to pleiotropy or tightly linkage for the three elements, especially Cd and As under treatment condition.

### Candidate gene analysis for 14 important QTL

**FIGURE 2:**
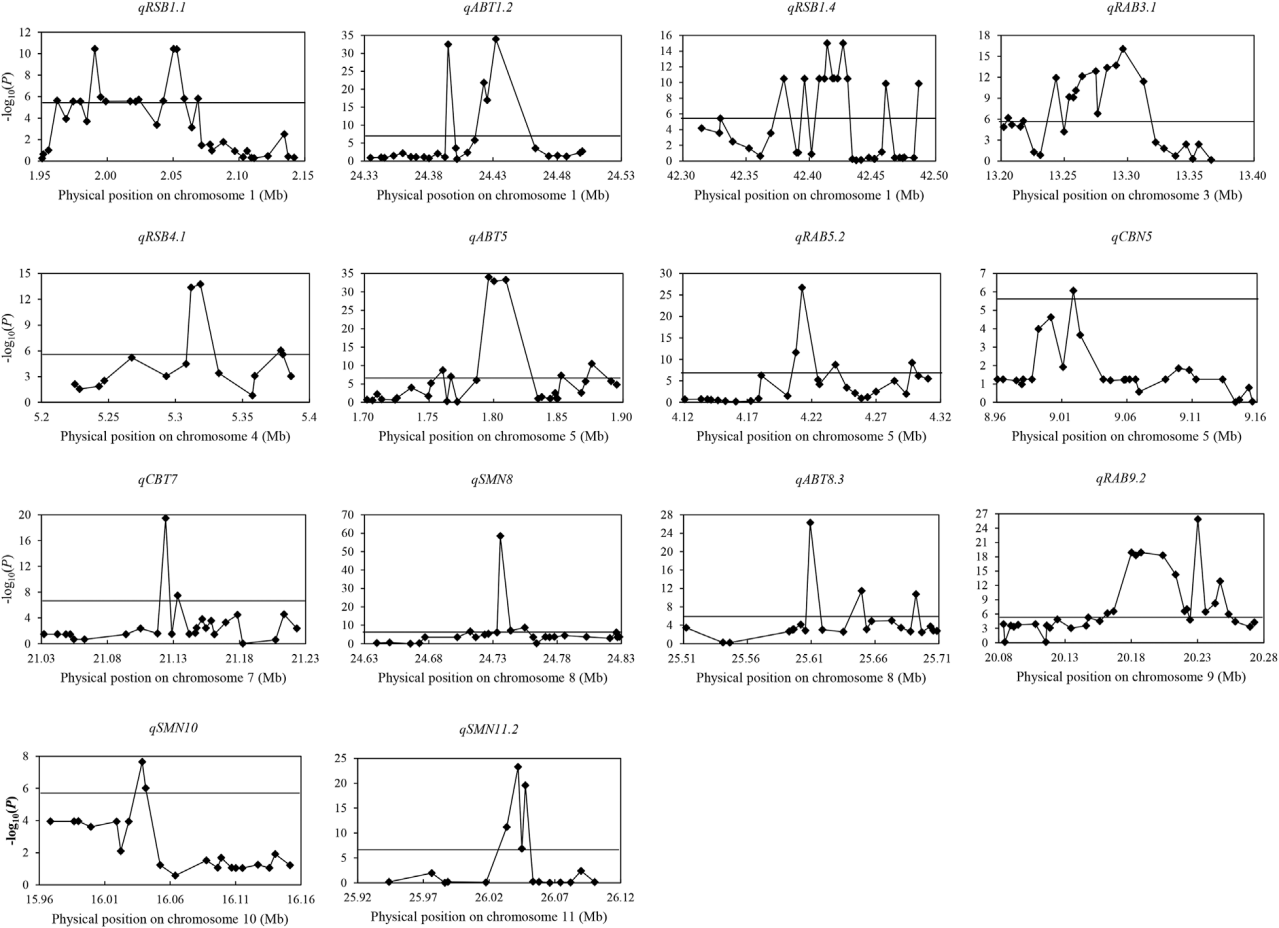
Manhattan plots of gene-based association analysis in 14 important QTL regions. The rhomb-shaped point represents annotation genes in the candidate region; the horizontal line represents the threshold.

Among 237 QTL for concentrations of three elements, 14 important QTL were identified in both years and/or had effect on more than two different traits. A total of 40 candidate genes and 43 favorable alleles were identified by gene-based association analysis and haplotype analysis (Table 2; Supplementary Table S6; Figure 2).

**TABLE 2:** Information of candidate genes in 14 important QTL regions.

QTL	Chr	Confidence interval (Mb)	MSU ID	Annotation	Trait affected
*qRSB1.1*	1	1.95–2.15	*LOC_Os01g04580*	Ser/Thr protein kinase, putative, expressed	RSB/RCM
			*LOC_Os01g04590*	expressed protein	RCM/SBT/RSB
*qABT1.2*	1	24.33–24.53	*LOC_Os01g42870*	transferase family protein, putative, expressed	ABT/SBT/CBT
			*LOC_Os01g42909*	hypothetical protein	ABT/CBT
			*LOC_Os01g42950*	protein kinase family protein, putative, expressed	ABT/CBT
*qRSB1.4*	1	42.31–42.51	*LOC_Os01g73040*	CBS domain-containing protein, putative, expressed	RSB/RCM/SBT
			*LOC_Os01g73080*	expressed protein	RSB/RCM/SBT
			*LOC_Os01g73110*	expressed protein	RSB/RCM/SBT
			*LOC_Os01g73120*	expressed protein	RSB/RCM/SBT
			*LOC_Os01g73130*	vacuolar ATP synthase, putative, expressed	RSB/RCM/SBT
			*LOC_Os01g73140*	ubiquitin-fold modifier 1 precursor, putative, expressed	RSB/RCM/SBT
			*LOC_Os01g73150*	expressed protein	RSB/RCM/SBT
			*LOC_Os01g73170*	peroxidase precursor, putative, expressed	RSB/RCM/SBT
			*LOC_Os01g73250*	abscisic stress-ripening, putative, expressed	RCM
*qRAB3.1*	3	13.20–13.40	*LOC_Os03g22840*	retrotransposon protein, putative, unclassified, expressed	RAB/ABT/RCB
*qRSB4.1*	4	5.22–5.42	*LOC_Os04g09880*	expressed protein	RSB/RCM
*qABT5*	5	1.69–1.89	*LOC_Os05g03934*	expressed protein	ABT/CBT
			*LOC_Os05g03972*	plant protein of unknown function domain containing protein, expressed	ABT/CBT/RCB/RAB
			*LOC_Os05g04000*	expressed protein	ABT/CBT/RCB/RAB
			*LOC_Os05g04020*	plant protein of unknown function domain containing protein, expressed	ABT/CBT/RAB
*qRAB5.2*	5	4.11–4.31	*LOC_Os05g07810*	universal stress protein domain containing protein, putative, expressed	RAB
*qCBN5*	5	8.95–9.15	*LOC_Os05g15960*	retrotransposon protein, putative, unclassified, expressed	CBN
*qCBT7*	7	21.02–21.22	*LOC_Os07g35290*	TKL_IRAK_DUF26-lc.10 - DUF26 kinases have homology to DUF26 containing loci, expressed	CBT/ABT/RAB
			*LOC_Os07g35310*	TKL_IRAK_DUF26-lc.12 - DUF26 kinases have homology to DUF26 containing loci, expressed	CBT/ABT/RCB/RAB
*qSMN8*	8	24.64–24.84	*LOC_Os08g39120*	expressed protein	SMN/SBN
			*LOC_Os08g39174*	retrotransposon protein, putative, Ty1-copia subclass, expressed	SMN/SBN
			*LOC_Os08g39180*	OsWAK73 - OsWAK receptor-like protein kinase, expressed	SMN/SBN
*qABT8.3*	8	25.50–25.70	*LOC_Os08g40510*	KID-containing protein, putative, expressed	ABT/CBT
			*LOC_Os08g40570*	pyridoxamine 5%27-phosphate oxidase family protein, putative, expressed	ABT/CBT/RCB
*qRAB9.2*	9	20.08–20.28	*LOC_Os09g34200*	OsFBX338 - F-box domain containing protein, expressed	RAB/CBT/ABT/RCB
			*LOC_Os09g34214*	UDP-glucoronosyl and UDP-glucosyl transferase domain containing protein, expressed	RAB/CBT/ABT/RCB
			*LOC_Os09g34230*	UDP-glucoronosyl/UDP-glucosyl transferase, putative, expressed	RAB/CBT/ABT/RCB
			*LOC_Os09g34250*	UDP-glucoronosyl and UDP-glucosyl transferase domain containing protein, expressed	RAB/CBT/ABT/RCB
			*LOC_Os09g34270*	UDP-glucoronosyl and UDP-glucosyl transferase domain containing protein, expressed	RAB/CBT/ABT/RCB
*qSMN10*	10	15.95–16.15	*LOC_Os10g30790*	inorganic phosphate transporter, putative, expressed	SMN/SBN/RAB
			*LOC_Os10g30800*	expressed protein	SMN/SBN/RAB
*qSMN11.2*	12	25.94–26.14	*LOC_Os11g43140*	expressed protein	SMN/SBN
			*LOC_Os11g43150*	transposon protein, putative, CACTA, En/Spm sub-class, expressed	SMN/SBN
			*LOC_Os11g43160*	ransposon protein, putative, CACTA, En/Spm sub-class	SMN/SBN
			*LOC_Os11g43170*	hypothetical protein	SMN/SBN

SBN, se concentration in brown rice in normal condition; SMN, se concentration in milled rice in normal condition; CBN, cd concentration in brown rice in normal condition; SBT, se concentration in brown rice in treatment condition; CBT, cd concentration in brown rice in treatment condition; ABT, as concentration in brown rice in treatment condition; RSB, ratio of Se concentration in brown rice; RCB, ratio of Cd concentration in brown rice; RCM, ratio of Cd concentration in milled rice; RAB, ratio of As concentration in brown rice. The QTL listed in Supplementary Table S4.

For *qRSB1.1* in 1.95–2.15 Mb on chromosome 1, 1,598 SNPs were found in 34 genes. Among them, 14 genes were identified. Significant differences were identified in RSB and RCM in 2019 between different haplotypes for two candidate genes (*LOC_Os01g04580* and *LOC_Os01g04590*). Also, there was significant difference in SBT in 2019 between two haplotypes in *LOC_Os01g04590*. No haplotype was found for the rest 12 genes.

For *qABT1.2* in the region of 24.33–24.53 Mb on chromosome 1, 449 SNPs were found in 25 genes, and five candidate genes were identified. Significant differences were detected in ABT and CBT in 2019 between different haplotypes in candidate genes *LOC_Os01g42870*, *LOC_Os01g42909* and *LOC_Os01g42950*. Besides, there was significant difference in SBT in 2019 between two haplotypes in *LOC_Os01g42870*. No haplotype was found for the other two genes (*LOC_Os01g42920* and *LOC_Os01g42934*).

For *qRSB1.4* in 42.31–42.51 Mb on chromosome 1, 1,137 SNPs were found in 34 genes, and 13 candidate genes were identified. Significant differences in RSB, SBT and RCM in 2019 were detected between two haplotypes in *LOC_Os01g73040*, *LOC_Os01g73080*, *LOC_Os01g73110*, *LOC_Os01g73120*, *LOC_Os01g73130*, *LOC_Os01g73140*, *LOC_Os01g73150* and *LOC_Os01g73170*. Besides, there was significant difference in RCM in 2019 between two haplotypes in *LOC_Os01g73250.* No haplotype was found for *LOC_Os01g72980*, *LOC_Os01g73100*, *LOC_Os01g73160* and *LOC_Os01g73310*.

For *qRAB3.1* in the region of 13.20–13.40 Mb on chromosome 3, 305 SNPs were found in 26 genes, and 14 candidate genes were identified. Significant differences were detected in RAB, ABT and RCB in 2019 between different haplotypes in *LOC_Os03g22840*. There was no significant difference in any trait between different haplotypes or no haplotype available in the rest 13 genes.

For *qRSB4.1* in the region of 5.22–5.42 Mb on chromosome 4, 818 SNPs were found in 15 genes, and five candidate genes were identified. Significant differences were detected in RSB and RCM in 2019 between two haplotypes in *LOC_Os04g09880*. There was no significant difference in any trait between different haplotypes or no haplotype identified in the rest four genes.

For *qABT5* in the region of 1.69–1.89 Mb on chromosome 5, 1,086 SNPs were found in 29 genes, and 11 candidate genes were identified. Significant differences were detected in ABT and CBT in 2019 between different haplotypes in *LOC_Os05g03934*, *LOC_Os05g03972*, *LOC_Os05g04000* and *LOC_Os05g04020*. Besides, significant differences in RCB and RAB in 2019 were found between two haplotypes of each of *LOC_Os05g03972* and *LOC_Os05g04000*, and there was also significant difference in RAB in 2019 between two haplotypes in *LOC_Os05g04020*. There was no significant difference in any trait between different haplotypes or no haplotype available in the rest seven genes.

For *qRAB5.2* in the region of 4.11–4.31 Mb on chromosome 5, 1,508 SNPs were found in 26 genes, and nine candidate genes were identified. Significant difference was detected in RAB in 2019 between two haplotypes in *LOC_Os05g07810*. No haplotype was found in the rest eight genes.

For *qCBN5* in the region of 8.95–9.15 Mb on chromosome 5, 596 SNPs were found in 28 genes, and only one candidate gene (*LOC_Os05g15960*) was identified. Significant difference was detected in CBN in 2019 between the two haplotypes.

For *qCBT7* in the region of 21.02–21.22 Mb on chromosome 7, 1,245 SNPs were found in 26 genes, and two candidate genes (*LOC_Os07g35290* and *LOC_Os07g35310*) were identified. Significant differences were detected in CBT, ABT and RAB in 2019 between two haplotypes in both the two genes. Besides, there was significant difference in RCB in 2019 between two haplotypes in *LOC_Os07g35310*.

For *qSMN8* in the region of 24.64–24.84 Mb on chromosome 8, 629 SNPs were found in 25 genes, and seven candidate genes were identified. Significant differences were detected in SMN and SBN in 2020 between two haplotypes in *LOC_Os08g39120*, *LOC_Os08g39174* and *LOC_Os08g39180.* No haplotype was found in *LOC_Os08g39150*, *LOC_Os08g39160*, *LOC_Os08g39170* and *LOC_Os08g39280*.

For *qABT8.3* in the region of 25.50–25.70 Mb on chromosome 8, 620 SNPs were found in 22 genes, and three candidate genes (*LOC_Os08g40470*, *LOC_Os08g40510* and *LOC_Os08g40570*) were identified. Significant differences were detected in ABT and CBT in 2019 between two haplotypes in *LOC_Os08g40510* and *LOC_Os08g40570.* Besides, significant difference was also found in RCB in 2019 between two haplotypes in *LOC_Os08g40570.* No haplotype was found in *LOC_Os08g40470*.

For *qRAB9.2* in the region of 20.08–20.28 Mb on chromosome 9, 1,162 SNPs were found in 32 genes, and 15 candidate genes were identified. Significant differences were detected in RAB, ABT, CBT and RCB in 2019 between different haplotypes in *LOC_Os09g34200*, *LOC_Os09g34214*, *LOC_Os09g34230*, *LOC_Os09g34250* and *LOC_Os09g34270*. There was no significant difference in any trait between different haplotypes or no haplotype available in the rest 10 genes.

For *qSMN10* in the region of 15.95–16.15 Mb on chromosome 10, 807 SNPs were found in 21 genes, and two candidate genes (*LOC_Os10g30790* and *LOC_Os10g30800*) were identified. Significant differences were detected in SBN and SMN in 2020, and RAB in 2019 between two haplotypes in both the two genes.

For *qSMN11.2* in the region of 25.94–26.14 Mb on chromosome 11, 376 SNPs were found in 16 genes, and four candidate genes (*LOC_Os11g43140, LOC_Os11g43150*, *LOC_Os11g43160* and *LOC_Os11g43170*) were identified. Significant differences were detected in SMN and SBN in 2020 between two haplotypes in all four candidate genes.

## Discussion

### Distribution characteristics of three elements in rice grains and Se-treatment effect on their concentrations

Most Chinese people are likely to consume white rice or polished rice which is produced from brown rice. Although rice bran only accounts for 6–10% of the total weight of rice (Wang et al., 2017), it contains 64% of important nutrients or toxic elements (Yang et al., 2004). Our research showed that the levels of toxic elements such as As and Cd, and nutritional element Se have a good linear relationship between rice bran and polished rice, and most of them are enriched in rice bran as indicated that SBN, CBN and ABN accounted for almost more than half of those in the whole grain. Relatively speaking, the proportion of Se concentration in polished rice to the whole grain is higher than that of Cd. The differences of element distribution in rice grains provide an effective way to remove most of toxic elements enriched in bran by polishing brown rice, thus better ensuring food security for people who consume the polished rice.

It was reported that the average Se concentration in rice grains is 0.025 mg kg^−1^ in different regions of China, which results in the Se deficiency of inhabitants in China because the rice Se is the main source of Se in the Chinese diet (Chen et al., 2002). Biofortification of rice using Se fertilizers not only increases grain Se concentration, but also reduces grain Cd concentration (Tan et al., 2014). This was supported by our finding that Se fertilizer treatment substantially improved Se concentrations but had no obvious changes in concentrations of Cd and As both in brown and milled rice in this study (Table 1). It is the first report in the present study that the reactions of Cd and As in rice grain to Se fertilizer treatment varied largely among rice accessions. Take the concentrations in milled rice as example, 26.51% and 20.11% accessions significantly decreased in Cd and As concentrations (RCM and RAM below 0.80) after Se treatment, 27.71% and 15.64% accessions significantly increased in Cd and As concentrations (RCM and RAM above 1.5), and 45.78% and 64.25% accessions were insensitive to Se treatment in Cd and As concentrations (RCM and RAM between 0.8 and 1.5), respectively. Thus, screening varieties whose Cd and As concentrations are insensitive to Se treatment or even decreased in milled rice after Se treatment would provide an effective way of biofortification for rice to enhance Se concentration and avoid enrichment of toxic elements through spraying Se fertilizer on leaf.

### Comparisons of important QTL regions with ones previously reported and their candidate gene inference

In the present study, a total of 14 important QTL regions was identified in both years and/or had effect on more than two different traits (Table 2). Most of them were identified in the same or adjacent regions with ones previously reported (Table 2; Supplementary Table S5). For example, *qRSB1.1*, *qSBT1.1* and *qRCM1.1* at the peak position of 2.05 Mb, *qRSB1.4*, *qSBT1.3* and *qRCM1.2* in the region of 42.25–42.60 Mb on chromosome 1 were identified in the same regions of *qCd1-2* for grain Cd concentration (Zhang G. M. et al., 2018) and *qGCD8* for grain Cd concentration (Liu et al., 2019), and *OsLCD* for Cd tolerance and accumulation (Chen et al., 2019). *qRAB3.1*, *qABT3.1* and *qRCB3.1* in the region of 13.16–13.30 Mb on chromosome 3 were detected together with *qGAS15* for grain As accumulation (Liu et al., 2019). *qRCM4.1*, and *qRSB4.1* in the 5.15–5.32 region on chromosome 4 were found near *qCd4-3* for grain Cd concentration (Zhang G. M. et al., 2018). *qABT5*, *qCBT5*, *qRCB5.1* and *qRAB5.1* in the region of 1.79–1.80 Mb, and *qRAB5.2* in the region of 4.21–4.33 Mb on chromosome 5 were identified in the same regions of *qSe5-1* for grain Se concentration (Zhang et al., 2010), *qCd5-1* for grain Cd concentration (Zhang G. M. et al., 2018), *OsMTP1* for Cd translocation, and *OsZIP6* for Cd transport (Chen et al., 2019). *qCBT7*, *qABT7*, *qRCB7.2* and *qRAB7* in the peak position of 21.12 Mb on chromosome 7 were detected near *qGAS13* for grain As accumulation (Liu et al., 2019) and *qCd7-2* for grain Cd accumulation (Zhang G. M. et al., 2018). *qABT8.3*, *qCBT8.2*, *qRCB8.3* and *qRAB8.3* at the peak position of 25.60 Mb on chromosome 8 were mapped together with *qCd8-3* for grain Cd concentration (Zhang G. M. et al., 2018). *qRAB9.2*, *qABT9.2*, *qCBT9.2* and *qRCB9.2* in the region of 20.18–20.19 Mb on chromosome 9 were detected near *qRCd-5* for grain Cd accumulation (Pan et al., 2021). Whether the QTL identified in this study are allelic to the previously reported QTL or genes need to be validated by fine-mapping and transgenic strategy.

The candidate genes were further inferred by bioinformatics and gene expression at filling or milk stage of grain. Heavy metal accumulation in rice grains may occur at grain filling or milk stage. To identify the candidate genes for QTL involved in this process, we analyzed the expression pattern of candidate genes for each QTL using the RNA-seq database from MBKBASE. For three candidate genes of *qABT1.2*, *LOC_Os01g42909*, encoding hypothetical protein, was specifically expressed at milk stage of embryo and endosperm (Supplementary Figure S3). For *qRSB1.4*, *LOC_Os01g73040* and *LOC_Os01g73130* encode CBS domain-containing protein and vacuolar ATP synthase, respectively. A rice gene (*OsCBSX4*) with CBS domain involved in heavy metal tolerance (Singh et al., 2012). Besides, some heavy metal ATPase, OsHMA2 and OsHMA3, had function on Cd concentrations in grains (Yan et al., 2019). *LOC_Os01g73140* encodes precursor of UFM1 (ubiquitin-fold modifier 1) and is highly expressed in the milk stage of grain and filling stage of endosperm (Supplementary Figure S4). UFM1 protein functions as new post-translational UBLs (ubiquitin-like proteins) with similar structure and regulatory mechanism with ubiquitin. Although the function of UFM1 has not been reported in plants, the human UFM1 participates in the ER (endoplasmic reticulum) stress response that may involve in the folding and transport of protein (Kang et al., 2007; Li et al., 2018), suggesting that *LOC_Os01g73140* may involve in the regulation of element content. So, *LOC_Os01g73040*, *LOC_Os01g73130* and *LOC_Os01g73140* are likely candidate genes for *qRSB1.4*. Of three candidate genes for *qSMN8*, *LOC_Os08g39120* encodes an unknown expressed protein, with higher expression level in the milk stage of grain and filling stage of endosperm compared with the other two genes (Supplementary Figure S5), suggesting that *LOC_Os08g39120* is the most likely candidate gene for *qSMN8*. For *qRAB9.2*, five candidate genes were identified, and *LOC_Os09g34200* encodes F-box domain proteins. In previous study, many F-box proteins were highly expressed at Cd stress (Zhou et al., 2012; Chen et al., 2014), implying that they may involve in Cd transport in plants and affect Cd accumulation. Another candidate gene, *LOC_Os09g34230*, encoding UDP-glucoronosyl/UDP-glucosyl transferase protein, is highly expressed at milk stage of grain and filling stage of endosperm, as well as mature stage of grain (Supplementary Figure S6), indicating that *LOC_Os09g34230* plays important roles in the rice grain ripening process. UDP-glucosyl transferase has been found to be involved in biologic and abiotic stress responses in wheat, *Arabidopsis* and *Rhazya stricta* (Hajrah et al., 2017; Sharma et al., 2018; Rao et al., 2019). The candidate gene *LOC_Os10g30790* for *qSMN10*, encoding inorganic phosphate transporter, is expressed at milk stage of grain, filling stage of endosperm, mature stage of grain and especially highly expressed at mature stage of aleurone that exists in brown rice (Supplementary Figure S7). Importantly, the expression of phosphate transporter could increase arsenic tolerance in *Arabidopsis* (Feng et al., 2021), indicating that *LOC_Os10g30790* is the most likely candidate gene for *qSMN10*, which mediates the response to arsenic stress. Above most likely candidate genes will be validated using CRISPR-Cas9 and transgenic technology in future.

### Application in rice breeding for Se enrichment with decreased Cd and As

In China, the general population in 72% of the total land area is facing a Se deficiency problem (Feng et al., 2013). Therefore, it is of great significance to develop rice variety with Se enrichment but minimum contents of heavy metals such as Cd and As in milled rice. Liu et al. (2020) reported that new elite varieties with enriched Se content in grains can be realized by pyramiding different main-effect QTL that considerably facilitated high Zn/Se enrichment while low Cd accumulation in grains. In the present study, among 142 QTL for concentrations of three elements detected in normal or treatment conditions (Supplementary Table S4), only 19 QTL (13.4%) were commonly detected for concentrations of Se and Cd, Se and As, and Se, Cd and As in the same or adjacent regions (Supplementary Table S5), indicating that most genetic loci underlying Se concentration are independent of those controlling Cd and As concentrations. Breeders could pyramid favorable alleles at different Se QTL and excluding undesirable alleles at Cd and As QTL, thus, simultaneously increase Se concentration and minimize Cd and As concentrations. Based on haplotype analysis of candidate genes in 14 important QTL regions identified in this study, Hap2 of candidate gene *LOC_Os08g39120* at *qSMN8*, *LOC_Os10g30790* at *qSMN10*, and Hap1 of *LOC_Os11g43140* or *LOC_Os11g43150*, or *LOC_Os11g43170* at *qSMN11.2* could increase Se concentration in both brown and milled rice. Three accessions, IRIS_313–8208, IRIS_313–8856 and IRIS_313–11968 identified with high Se and low Cd and As concentrations in milled rice carried 2, 3 and 1 favorable alleles of the candidate genes at the three loci underlying Se concentration, respectively (Supplementary Table S7). Meanwhile, the three accessions all carried favorable Hap 1 of *LOC_Os05g15960* at *qCBN5* which could decrease CBN. So, the three accessions could be used as donor parents to introgress then pyramid different favorable alleles at the three QTL for Se concentration and one QTL for Cd concentration by marker-assisted selection (MAS).

In most cases, Se deficiency can be corrected by the application of Se fertilizer into the soils or on rice leaf, which is termed as agronomic biofortification (Lyons et al., 2004). As indicated in this study, Se fertilizer treatment substantially improved Se concentrations but had no obvious changes in concentrations of Cd and As both in brown and milled rice, meaning as compared with Se, Cd and As in grains were insensitive to Se fertilizer treatment. Three important QTL regions (1.95–2.15 Mb and 42.31–42.51 Mb on chromosome 1, and 5.22–5.42 Mb on chromosome 4) were identified for both RSB and RCM in this study, and Hap1 of *LOC_Os01g04580* at *qRSB1.1*/*qRCM1.1* and *LOC_Os04g09880* at *qRSB4.1/qRCM4.1*, and Hap2 of *LOC_Os01g04590* at *qRSB1.1*/*qRCM1.1*, *LOC_Os01g73040*, *LOC_Os01g73130* and *LOC_Os01g73140* at *qRSB1.4*/*qRCM1.2* could enhance about 5.1–31.6 times of the Se concentration in brown rice and 5.4–10.5 times of Cd concentrations in milled rice after Se treatment. So, introgressing and pyramiding favorable genes for above three QTL could much improve Se concentration in grains after Se fertilizer treatment although Cd concentration could be increased to some extent due to synergistic effect of Se at these RSB QTL. Four accessions (CX115, IRIS_313–11039, IRIS_313–11197 and IRIS_313–11943) with high RSM and low RCM and RAM carried all favorable alleles at above QTL (Supplementary Table S7). Therefore, to develop higher Se-rich variety with more efficient response to Se treatment, above four accessions could be used as donor parent for introgressing and pyramiding of favorable alleles at *qRSB1.1*/*qRCM1.1*, *qRSB1.4*/*qRCM1.2* and *qRSB4.1/qRCM4.1* by MAS. It is worth noting that *qRSB3.2*/*qRSM3.1* for both RSB and RSM is only one locus which is independent of QTL for ratio of Cd and As concentrations. So, after mining favorable alleles at this locus using more diverse germplasms, the favorable alleles can be used for further enhancing Se concentrations in both brown and milled rice after Se treatment without interference of Cd and As in rice breeding for Se biofortification.

Finally, another efficient strategy to enhance Se concentration in rice grains is probably to pyramid favorable alleles of different kinds of QTL, i.e., QTL for Se enrichment identified in normal condition and QTL for Se sensitivity identified under Se fertilizer treatment by MAS. Thus, new variety pyramiding above two kinds of QTL will show high Se concentration in normal condition and more higher Se concentration after Se fertilizer application in some Se-deficient areas.

## Conclusion

Large variations in concentrations of Se, Cd and As in grains existed in the panel of 307 rice accessions in normal and Se treatment conditions. Se fertilizer treatment greatly improved Se concentrations but had no obvious changes in concentrations of Cd and As both in brown and milled rice. A total of 237 QTL were identified for Se, Cd and As concentrations in normal, Se treatment and ratio of treatment to normal conditions by GWAS. Most QTL for Se concentration is independent of those for Cd and As concentrations in view of only 13.4% QTL commonly detected in the same or adjacent regions. Forty-three favorable alleles were identified for 40 candidate genes in 14 important QTL regions. Pyramiding of favorable alleles at Se QTL and excluding undesirable alleles at Cd and As QTL, or combining favorable alleles at Se QTL detected in normal condition with the alleles at Se-sensitive QTL detected under Se treatment by MAS will facilitate development of rice variety with Se enrichment and minimum concentrations of Cd and As.

## Data Availability

Publicly available datasets were analyzed in this study. This data can be found here: http://snp-seek.irri.org.
