# Supplementary material for: Distribution characteristics of selenium, cadmium and arsenic in rice grains and their genetic dissection by genome-wide association study
Source: Front Genet. 2022 Oct 13;13:1007896. doi: 10.3389/fgene.2022.1007896 (PMC9612882; doi:10.3389/fgene.2022.1007896)
Supplement: Supplementary file 3 [file Presentation1.PPTX]

## Slide 1
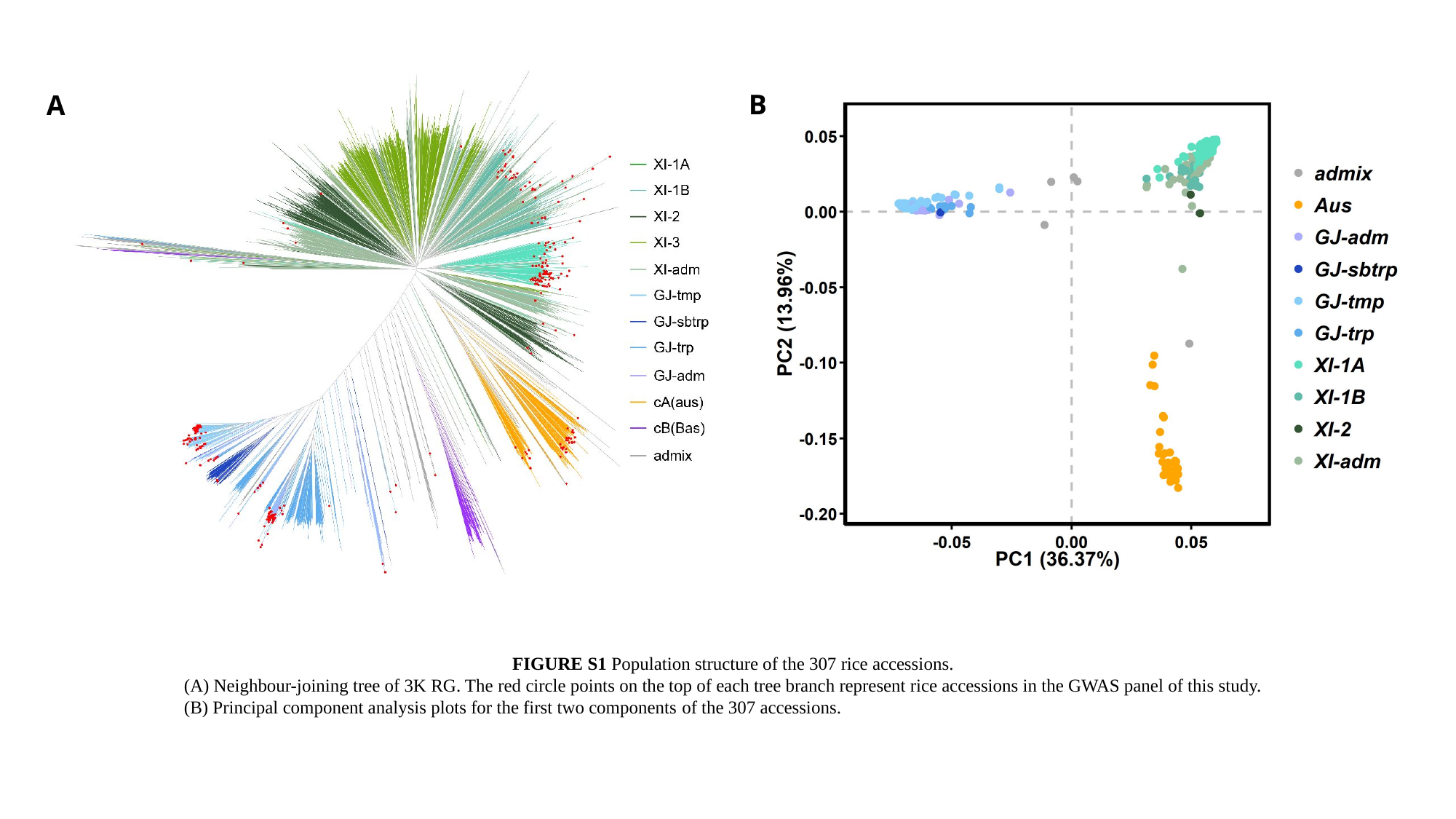

B
A
FIGURE S1 Population structure of the 307 rice accessions.
(A) Neighbour-joining tree of 3K RG. The red circle points on the top of each tree branch represent rice accessions in the GWAS panel of this study. (B) Principal component analysis plots for the first two components of the 307 accessions.
