# Supplementary material for: Distribution characteristics of selenium, cadmium and arsenic in rice grains and their genetic dissection by genome-wide association study
Source: Front Genet. 2022 Oct 13;13:1007896. doi: 10.3389/fgene.2022.1007896 (PMC9612882; doi:10.3389/fgene.2022.1007896)
Supplement: Supplementary file 10 [file Presentation2.PPTX]

## Slide 1
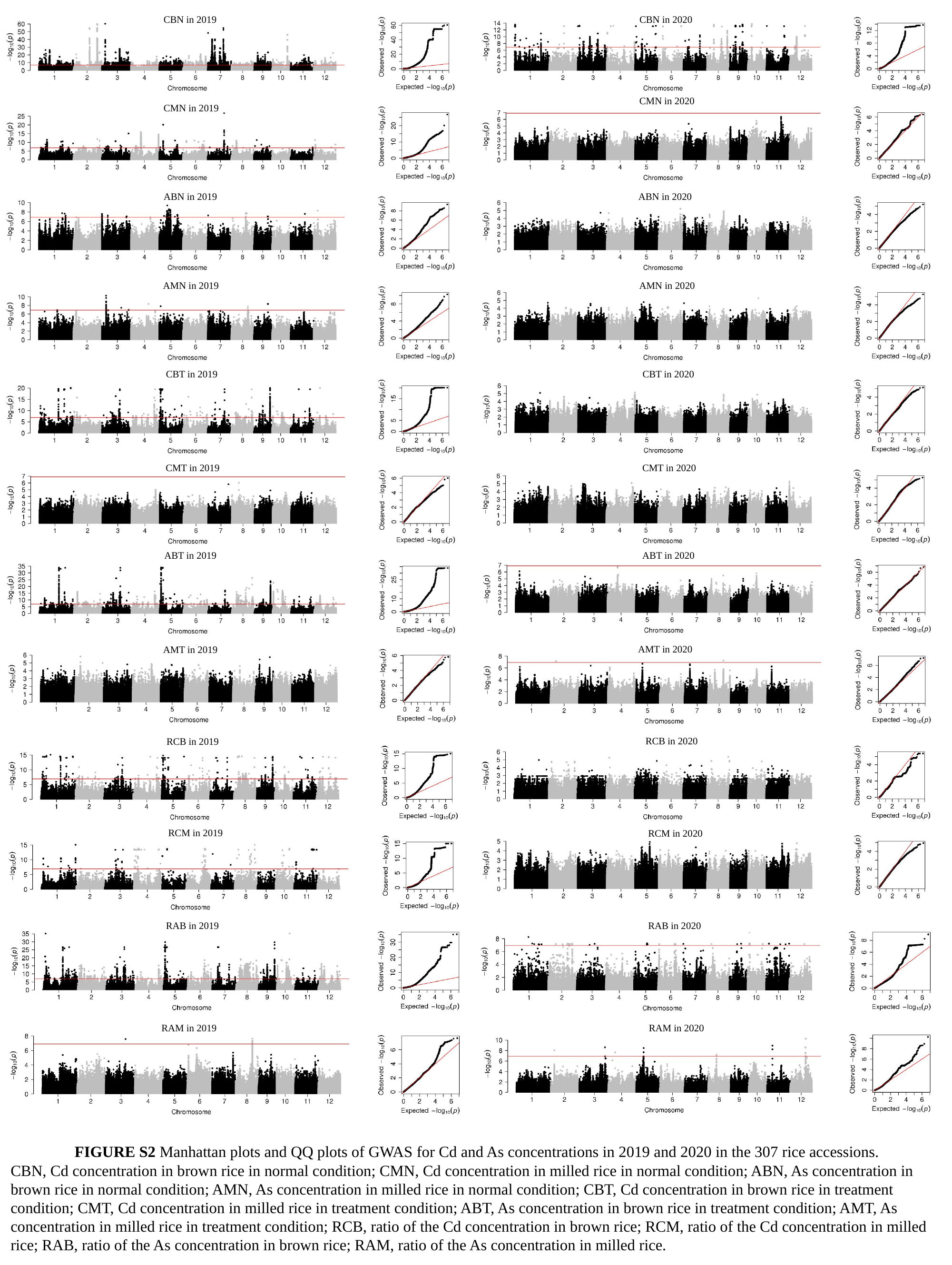

CBN in 2019
CBN in 2020
CMN in 2020
CMN in 2019
ABN in 2019
ABN in 2020
AMN in 2019
AMN in 2020
CBT in 2019
CBT in 2020
CMT in 2019
CMT in 2020
ABT in 2019
ABT in 2020
AMT in 2020
AMT in 2019
RCB in 2020
RCB in 2019
RCM in 2019
RCM in 2020
RAB in 2020
RAB in 2019
RAM in 2020
RAM in 2019
FIGURE S2 Manhattan plots and QQ plots of GWAS for Cd and As concentrations in 2019 and 2020 in the 307 rice accessions.
CBN, Cd concentration in brown rice in normal condition; CMN, Cd concentration in milled rice in normal condition; ABN, As concentration in brown rice in normal condition; AMN, As concentration in milled rice in normal condition; CBT, Cd concentration in brown rice in treatment condition; CMT, Cd concentration in milled rice in treatment condition; ABT, As concentration in brown rice in treatment condition; AMT, As concentration in milled rice in treatment condition; RCB, ratio of the Cd concentration in brown rice; RCM, ratio of the Cd concentration in milled rice; RAB, ratio of the As concentration in brown rice; RAM, ratio of the As concentration in milled rice.
