# Supplementary material for: Distribution characteristics of selenium, cadmium and arsenic in rice grains and their genetic dissection by genome-wide association study
Source: Front Genet. 2022 Oct 13;13:1007896. doi: 10.3389/fgene.2022.1007896 (PMC9612882; doi:10.3389/fgene.2022.1007896)
Supplement: Supplementary file 14 [file Presentation5.PPTX]

## Slide 1
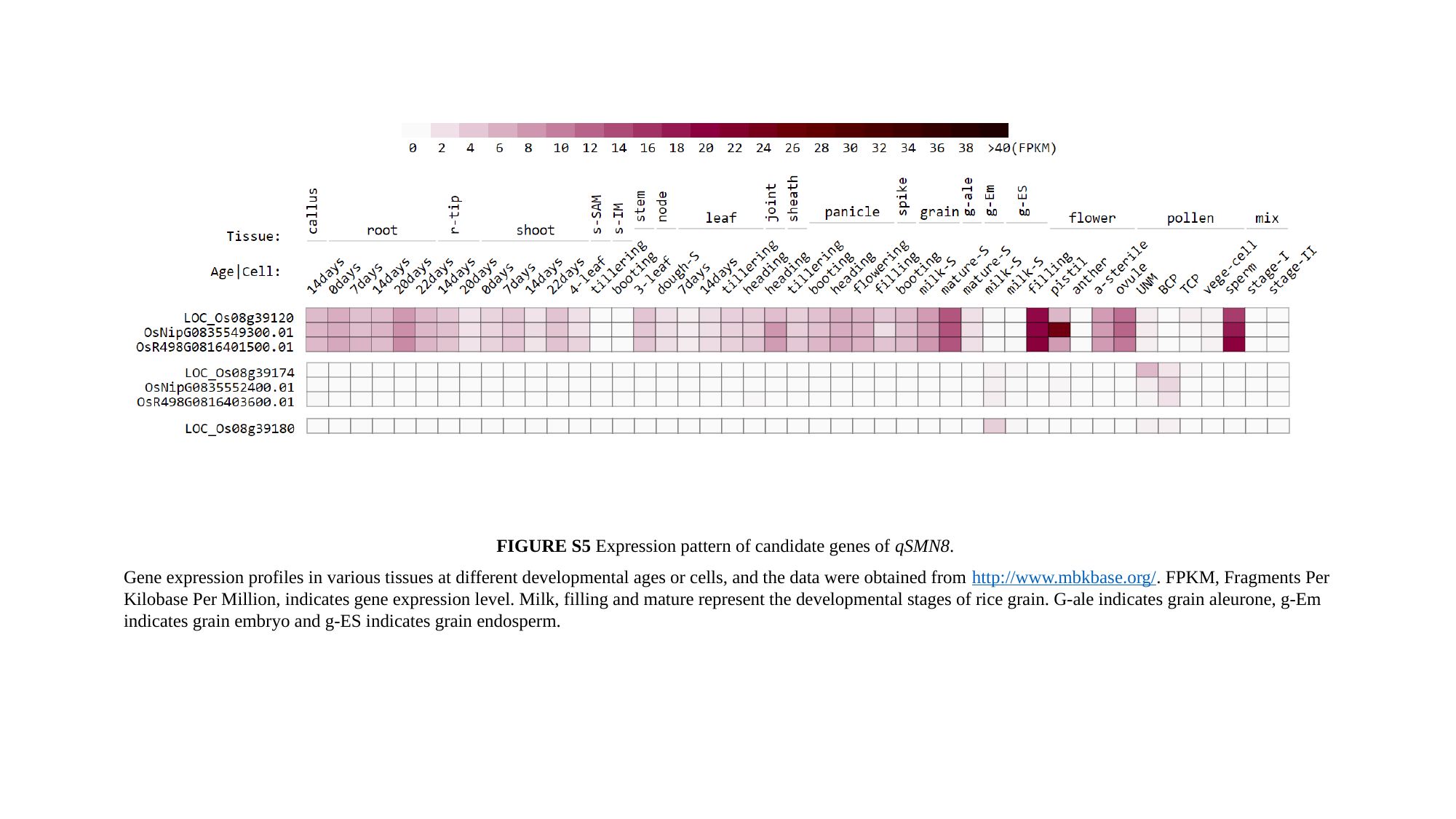

FIGURE S5 Expression pattern of candidate genes of qSMN8.
Gene expression profiles in various tissues at different developmental ages or cells, and the data were obtained from http://www.mbkbase.org/. FPKM, Fragments Per Kilobase Per Million, indicates gene expression level. Milk, filling and mature represent the developmental stages of rice grain. G-ale indicates grain aleurone, g-Em indicates grain embryo and g-ES indicates grain endosperm.
